# Infantile Fibrosarcoma With Concurrent Infantile Hemangioma: A Case Report

**DOI:** 10.7759/cureus.49586

**Published:** 2023-11-28

**Authors:** Nathan M Blair, Kristen G Berrebi, Deborah S Kacmarynski

**Affiliations:** 1 Pediatric Anesthesia, Departments of Anesthesiology and Pediatrics, University of Iowa Hospitals and Clinics, Iowa City, USA; 2 Pediatric Dermatology, Departments of Dermatology and Pediatrics, University of Iowa Hospitals and Clinics, Iowa City, USA; 3 Pediatric Otolaryngology, Department of Otolaryngology, Head and Neck Surgery, University of Iowa Hospitals and Clinics, Iowa City, USA

**Keywords:** pediatric otolaryngology, otolaryngology-head & neck surgeons, pediatrics, anesthesia considerations, pediatric anesthesia, pediatric dermatology

## Abstract

This report focuses on the clinical course and treatment of an infant male who had a progressively enlarging tongue mass initially thought to be an infantile hemangioma but was later found to be an infantile fibrosarcoma. Treatment included surgical excision with anticipated difficult mask ventilation with active rhino/enterovirus infection bronchiolitis and recent croup. Complete surgical excision is the mainstay of treatment, and the patient did have negative margins after complete surgical re-excision. The patient has surveillance MRI scans and remains without fibrosarcoma recurrence. This case report highlights complex pediatric airway management and the need for vigilance in healthcare when common presentations, such as infantile hemangioma, also present with a concurrent rare disease at a different anatomic location, such as infantile fibrosarcoma in this case.

## Introduction

The tongue is a complex organ with many important functions, including speech, mastication, and deglutition. Congenital masses of the tongue are most commonly benign vascular infantile hemangiomas, and these are also the most common soft tissue tumors in childhood [1]. Although rare, when a tongue mass does not respond to conventional treatments for hemangioma, prompt additional evaluation is needed to minimize patient risk from other potential benign and malignant tumors. As in this case, the patient presented with both a common infantile hemangioma in another location and a concurrent rare fibrosarcoma of the tongue. We will also describe the treatment and prognosis of tongue fibrosarcoma in an infant.

## Case presentation

**Figure 1:**
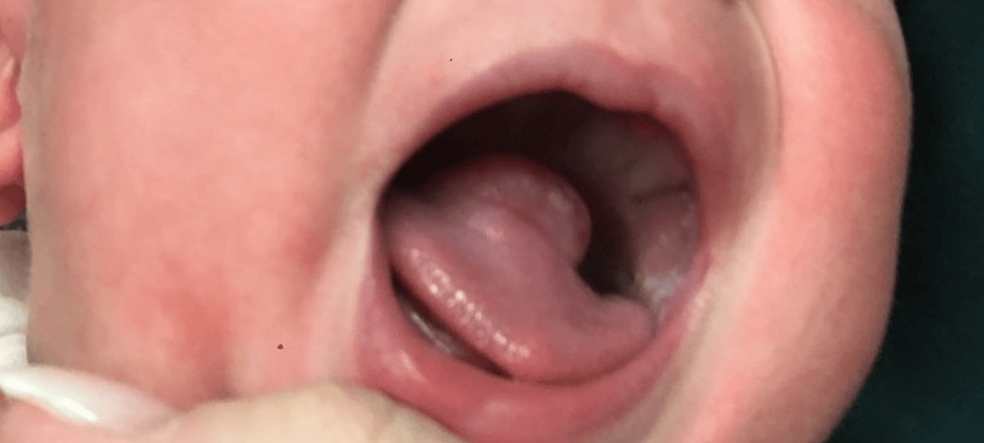
Tongue Mass

**Figure 2:**
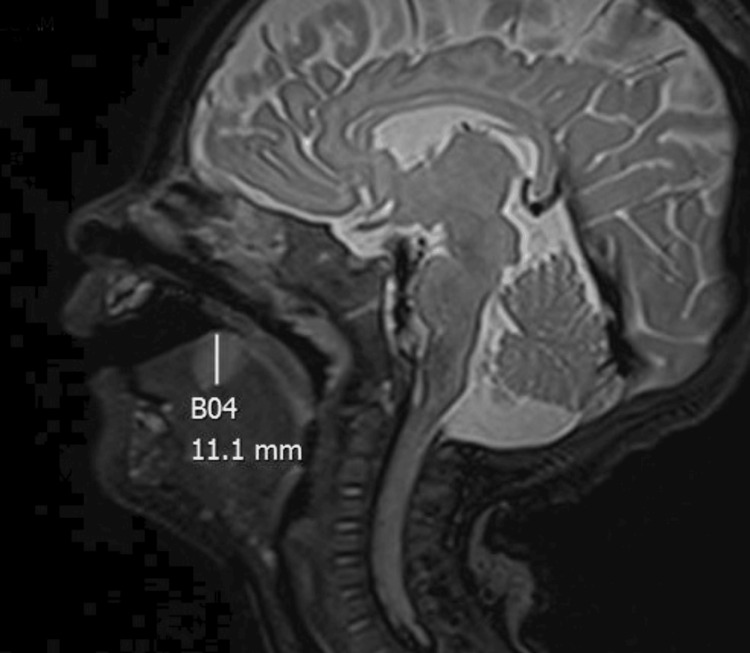
MRI with contrast demonstrating contrast enhancing 1.5 x 1.1 cm mid-tongue mass

A six-month-old male presented with a three-month history of enlarging right middle-third tongue mass, causing intermittent choking and gagging. The patient was born prematurely at 34 weeks due to pre-eclampsia, was treated with continuous positive airway pressure (CPAP) in the neonatal intensive care unit (NICU) for a short time after birth, and did not require intubation. In addition to the tongue mass, the patient also presented with an abdominal wall infantile hemangioma and recurrent croup. Due to similar growth timelines for both the abdominal wall hemangioma and tongue mass (Figure 1), the tongue mass was also thought to be an infantile hemangioma. Magnetic resonance imaging (MRI) of the head and neck supported this clinical suspicion and demonstrated a contrast-enhancing 1.5 x 1.1 cm right mid-tongue mass (Figures 1, 2) consistent with infantile hemangioma. Thus, the patient was started on oral propranolol therapy to treat both the abdominal hemangioma wall as well as the presumed tongue hemangioma. However, after taking oral propranolol for at least two months, the tongue mass was noted to have continued growth while, in contrast, the abdominal wall infantile hemangioma improved.

Due to concern regarding the continued growth of the tongue mass despite propranolol treatment, the patient was taken to the operating room (OR) for incisional biopsy versus excision. The patient had standard American Society of Anesthesiologists (ASA) monitors placed and then had a slow inhalational induction with spontaneous ventilation with sevoflurane and 100% oxygen. Intravenous access was placed with ultrasound. Possible difficult mask ventilation and difficult intubation were anticipated, and difficult airway equipment was prepared. He was nasally intubated successfully on the first attempt with a video laryngoscope Miller 1 blade. Backup airway plans included using a nasopharyngeal airway and having the surgeon place a tongue stitch to elevate the tongue in case of airway obstruction and difficult mask ventilation. The surgery was performed by marking a 5 mm boundary around the perimeter and excising with electrocautery using palpation to take a 5 mm cuff around the entire firm, irregular mass. The 2.0 x 2.5 cm mass was evaluated with preliminary pathology, giving an intraoperative diagnosis of spindle cell tumor, then a preliminary diagnosis of spindle cell rhabdomyosarcoma with initial positive myogenin on immunohistochemistry. The wound was closed with resorbable sutures in multiple layers, attempting to normalize the shape and volume of the mid-tongue. While in the OR, the patient did have bronchospasm, likely related to a recent upper respiratory infection (URI), that responded well to propofol, albuterol, and epinephrine. The patient was extubated in the OR and recovered in the pediatric intensive care unit (PICU) with a nasal pharyngeal airway and tongue stitch placed by the surgeon to help with airway obstruction during the immediate postoperative period. Post-operatively, the patient had increased work of breathing and oxygen desaturation and was found to have a superimposed rhino/enterovirus bronchiolitis that improved with racemic epinephrine and supplemental oxygen as needed. A nasogastric tube was placed for nutrition.

Staging workup computed tomography (CT)/positron emission tomography (PET) showed no distant metastatic disease. The tongue mass did have a positive margin and, therefore, was re-excised and closed in the OR seven days after the first surgery with negative frozen margins and no tumor found in this specimen; the patient was taken back to the PICU intubated with a nasal trumpet placed. No endotracheal tube cuff leak was appreciated two days later, despite decadron 0.5 mg/kg every six hours. A small cuff leak was present a day later, and the patient was taken to the OR for controlled extubation, where the cuff of the endotracheal tube had partially herniated at the level of the true vocal cords, creating a tight seal with the vocal cords and the deflated cuff balloon. 

The final pathology report, including expert outside consultation and molecular studies, was consistent with infantile fibrosarcoma with ETV6-NTRK3 gene fusion. Serial MRI neck imaging was obtained every quarter and then on an annual basis to assess for local recurrence. 

## Discussion

This six-month-old, ex-34-week patient presented with a tongue mass originally thought to be an infantile hemangioma, given its appearance, MRI findings, and an abdominal wall infantile hemangioma. Given the risk of bleeding from this location and the mass effect, the patient was considered at high risk for airway obstruction and difficult mask ventilation and intubation. We followed the described difficult airway algorithm, including using a video laryngoscope, having an available fiberoptic scope, and a difficult airway cart for case preparation [2]. The backup airway plans included using a nasopharyngeal airway and having the surgeon place a tongue stitch to help relieve airway obstruction from the tongue mass and help with difficult mask ventilation. We also decided against using a supraglottic airway and oral airways to minimize the mass's assumed bleeding risk. 

A complicating facet of this case included concurrent rhino/enterovirus infection bronchiolitis and recent croup (laryngotracheobronchitis). The nature and location of the expanding tongue mass did not allow time for the resolution of the airway inflammation before surgery. The most common cause of recurrent croup is a viral infection with the parainfluenza virus. In general, the leading causes of recurrent croup include anatomic airway abnormality (laryngomalacia, subglottic stenosis, tracheal stenosis, vascular rings, and others) and functional (asthma, gastroesophageal reflux disease, and eosinophilic esophagitis) [3]. The subglottis is the primary site of airway obstruction as this is the site of the narrowest portion of the pediatric airway, the cricoid cartilage. As the pediatric airway is already narrow, a small change in internal diameter due to swelling will have a significant change in airway resistance. We assumed, in this case, there was no endotracheal tube cuff leak due to airway edema from croup, when in fact, the cuff had partially herniated above the vocal cords while in the ICU and created a tight seal with the vocal cords even when the cuff was deflated. This was confirmed under direct visualization of the partially herniated cuff during the controlled extubation in the OR. The patient was then returned to the ICU for recovery.

Initial surgical pathology was consistent with spindle cell rhabdomyosarcoma, although the subsequent molecular studies did demonstrate an ETV6-NTRK3 gene fusion, a t(12;15)(p13;q25) rearrangement, highly specific (87.2%) for infantile fibrosarcoma [4]. Infantile fibrosarcoma is a very rare spindle-cell soft tissue sarcoma that is present at birth in 36% of affected patients, and the majority are less than one year of age [5]. Infantile fibrosarcoma is the most common sarcoma of infants less than one year of age [4]. The primary location of most infantile fibrosarcoma is frequently the extremities, often affecting the lower extremities more than the upper extremities [5]. Infantile fibrosarcoma may also affect the head and neck (more common in infants), although rarely affecting the tongue [6]. There are only a few case reports describing tongue infantile fibrosarcoma, and this case report adds to the sparse literature. 

The histomorphology of infantile fibrosarcoma and spindle cell rhabdomyosarcoma overlap; molecular studies and immunochemistry are essential to differentiate these tumors. The differentiation is critical for treatment and prognosis, as rhabdomyosarcoma requires chemotherapy. Microscopically, the tumor appears as spindle-shaped cells. The final immunohistochemical staining was positive for SMA, desmin, MYOD1, and pan-TRK. The apparent staining of desmin and MYOD1 is difficult to reconcile as this is either tumor cell staining or staining of entrapped reactive skeletal muscle fibers of the tongue [7].

Infantile fibrosarcoma has high local aggressiveness but overall favorable survival [4]. Surgical en bloc resection with clear margins is the mainstay of treatment for infantile fibrosarcoma, although only if no long-term functional or cosmetic impairments are anticipated [4]. No further treatment is recommended if clear margins are obtained, as this is generally considered curative. Close surveillance with MRI is recommended. If surgery is deemed unacceptable either functionally or cosmetically, then chemotherapy with vincristine-actinomycin-D is initiated until surgical resection is feasible [4].

## Conclusions

Common presentations, such as infantile hemangioma, may also present with a concurrent rare disease, such as infantile fibrosarcoma; thus, vigilance is critical to decipher between these common and uncommon diagnoses. This case was additionally challenging given the potential for difficult mask ventilation and difficult intubation due to the size and location of the tongue mass with active lower respiratory tract infection. The patient underwent complete surgical excision of the tongue fibrosarcoma and remains without recurrence. 
